# The prevalence of osteoporotic fractures in the elderly in China: a systematic review and meta-analysis

**DOI:** 10.1186/s13018-023-04030-x

**Published:** 2023-07-27

**Authors:** Shilong Meng, Minghao Tong, Yang Yu, Yanguang Cao, Binbin Tang, Xiaolin Shi, Kang Liu

**Affiliations:** 1 The Second School of Clinical Medicine, Zhejiang Chinese Medical University, Hangzhou, Zhejiang Province China; 2 Orthopedic Traumatology II, The Second Affiliated Hospital of Zhejiang Chinese Medical University, Hangzhou, China

**Keywords:** Osteoporotic fractures, Elderly, Chinese, Prevalence, Meta-analysis

## Abstract

**Background:**

Prevalence information is the first step in developing preventive procedures or health services. This study was conducted to systematically evaluate the epidemiology of osteoporotic fractures in Chinese elderly aged ≥ 60 years and to provide evidence-based evidence for the prevention and treatment of osteoporotic fractures.

**Methods:**

We identified relevant studies by searching the literature published in PubMed, Web of Science, Cochrane Library, Embase, CNKI, Wanfang Data, and VIP databases from the establishment of the database until August 2022. We used a random-effects model to obtain prevalence estimates and identified sources of heterogeneity and comparisons of prevalence among different groups through subgroup analysis and sensitivity analysis.

**Results:**

A total of 29 articles were included in this study, and the prevalence of osteoporosis fractures in elderly Chinese was high (18.9%). The prevalence has increased significantly over the past decade (from 13.2% in 2000–2010 to 22.7% in 2012–2022). The prevalence of osteoporosis is higher in women than in men (18.5% vs 14.3%) and increases with age. The northern region was higher than the southern region (20.3% vs 18.9%), and the spine, hip, and distal forearm were the most common sites of fracture.

**Conclusion:**

The prevalence of osteoporotic fractures in the Chinese elderly is 18.9%, and timely prevention and treatment are necessary.

**Supplementary Information:**

The online version contains supplementary material available at 10.1186/s13018-023-04030-x.

## Introduction

Osteoporotic fracture is a fracture caused by a low-energy external force, which is a serious consequence of osteoporosis [1]. Osteoporosis can lead to decreased bone strength and increased bone fragility, also known as fragility fractures. The high-risk groups for osteoporotic fractures are mainly the elderly and postmenopausal women. Fractures are mainly in important parts such as the lumbar spine, thoracic spine, and hip joints, and there is a risk of further fractures [2].

Osteoporotic fracture is a significant problem in the field of public health the world [3, 4]. It not only seriously affects the physical and mental health of elderly patients but also causes a substantial economic burden to the family and society [5–7]. The mortality and disability rate of osteoporotic fractures is very high. Studies have shown that the mortality rate of some elderly patients in the first year after hip fracture is as high as 17.1–33.0% [8, 9]; the 4-year mortality rate of conservative treatment of osteoporotic vertebral compression fractures (OVCF) can also be as high as 49.4% [10]. The number of patients with osteoporotic fractures is also huge. According to the International Osteoporosis Foundation [11], among middle-aged and older people over 50 years old, about 1/2 of women and 1/5 of men will experience at least one osteoporotic fracture, and 50% of patients may also have secondary fractures. Chinese scholars predict that [12]: it is expected that by 2035, China, will add about 4.83 million cases of osteoporotic fractures; approximately 5.99 million new topics will be added in 2050. The expenses for osteoporotic fractures in significant parts of the Chinese medical system will increase to 132 billion RMB and 163 billion RMB in 2035 and 2050, respectively.

Information on the prevalence of osteoporotic fractures in older adults is the first step in developing preventive procedures or health services for older adults. The problem of our study is the prevalence of osteoporotic fractures in Chinese ≥ 60 year olds. By systematically evaluating the epidemiology of osteoporotic fractures in Chinese ≥ 60 year olds, we can provide evidence-based evidence for the prevention and treatment of osteoporotic fractures.

## Methods

### Search strategy

This systematic review and meta-analysis were designed, conducted, and reported by the preferred reporting items for systematic reviews and meta-analyses (PRISMA) guidelines [13]. The ID number registered on Prospero is CRD42023383566. The following algorithm guided the preliminary search:

- Search strategy
- P (population): ≥ 60-year-old Chinese seniors
- I (intervention): no interventions
- C (comparisons): no comparisons
- (outcomes): prevalence of osteoporotic fractures in older Chinese people
- S (study): cross-sectional study

### Literature search

**Fig. 1:**
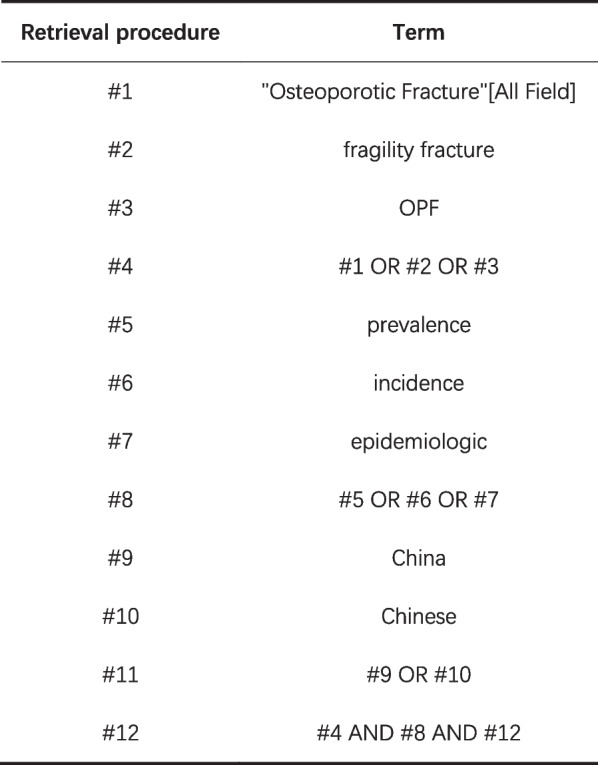
PubMed retrieval formula

The literature search process was conducted by two authors (SL.M; MH.T) independently conducted and the search process started in August 2022. We searched PubMed, Web of Science, Cochrane Library, Embase, CNKI, Wan Fang Data, and VIP database from establishing the database to August 1, 2022. The “subject word + free word” method was used for retrieval. Chinese search terms: “osteoporotic fracture”, “brittle fracture”, “low energy fracture”, “morbidity”, “prevalence”, “epidemiology”; English search terms: “Osteoporotic Fractures”, “OPF”, “fragility fracture”, “prevalence”, “incidence”, “epidemiology”, “China”, “Chinese”. According to the authors’ language capabilities, language of publication was restricted to English and Chinese. The PubMed search formula is as follows ((((Osteoporotic Fractures[All Fields]) OR (fragility fracture[All Fields]))) OR (OPF[All Fields])) AND (((prevalence[All Fields]) OR (epidemiologic[All Fields])) OR (incidence[All Fields]))) AND ((Chinese[All Fields]) OR (China[All Fields])). The literature retrieval process takes PubMed as an example, See Fig. 1.

### Study inclusion and exclusion criteria

Inclusion criteria: (1) According to the authors’ language capabilities, articles in English, Chinese were eligible. (2) Epidemiological study of osteoporotic fractures in China or Chinese population. (3) The study group included people aged 60 years and older. (4) The prevalence of osteoporotic fractures can be calculated. (5) Cross-sectional study.

Exclusion criteria: (1) The full text or result data cannot be obtained and needs to be completed or missing. (2) The same study was published in different journals. (3) The sample size of the elderly was too small. (≤ 100) (4) Research results published in the form of review, conference summary, expert consensus, etc.

### Data extraction and outcomes of interests

The retrieved literature was imported into EndNote software. Firstly, the duplicate literature was screened out, the title and abstract were read, and the research unrelated to the research problem was eliminated. Finally, the remaining literature was read in full text, included, and destroyed according to the inclusion and exclusion criteria. In this process, two evaluators (S-L.M. and M-H.T.) repeatedly cross-checked the included literature. A dispute will be resolved by discussing it between the two parties or introducing the third evaluator (Y.Y) for review.

The primary data extracted are as follows: (1) Basic information included in the literature: author, year of publication, survey area, etc. (2) Calculate the relevant data of the prevalence of osteoporotic fractures (Case Size, Sample Size, etc.); (3) critical information of bias risk assessment.

### Methodological quality assessment

The literature quality evaluation criteria proposed by KHAMBALIA, SEEN [14] were used to evaluate and record the quality of the included literature, as shown in Table 1.

**Table 1:** Quality evaluation criteria of cross-sectional literature

	Literature quality evaluation criteria	Score
1	A national epidemiological survey report with a large sample size (≥ 10,000) using a random sampling method	1
2	A provincial epidemiological survey report with a large sample size (≥ 1000) using a random sampling method	2
3	An epidemiological report on a limited number of specific units (e.g., 2 or 3 county-level cities or institutes) sampled randomly	3
4	Reports with a large sample size (≥ 1000) and without random sampling	4
5	Reports with large sample size (< 1000) but without random sampling	5

### Statistical analysis

We used Stata17.0 software to analyze the prevalence data. The classification of heterogeneity depends on I^2^ statistics [15]: < 25% indicates low level, 25–50% indicates moderate level, and > 50% indicates high heterogeneity. We used a random effects model to estimate the prevalence of osteoporotic fractures, five subgroups were set up: gender (male and female), age group (60–69, 70–79, ≥ 80), region (South, North), publication time (2000–2010, 2012–2022), and fracture site (vertebra, hip, distal forearm, and others). Sensitivity analysis was performed by eliminating references one by one, and statistical significance P-values were set at 0.05 in all statistical analyses. In this study, we did not examine publication bias. Publication bias refers to the fact that studies with significant results are more likely to be published than studies with non-significant results, which can lead to systematic differences between published and unpublished studies [16]. However, observational studies of prevalence do not have significant or negligible results, and it is not recommended to use mature methods to test for this bias in systematic reviews of prevalence studies. Therefore, we did not examine publication bias.

## Results

### Search result

**Fig. 2:**
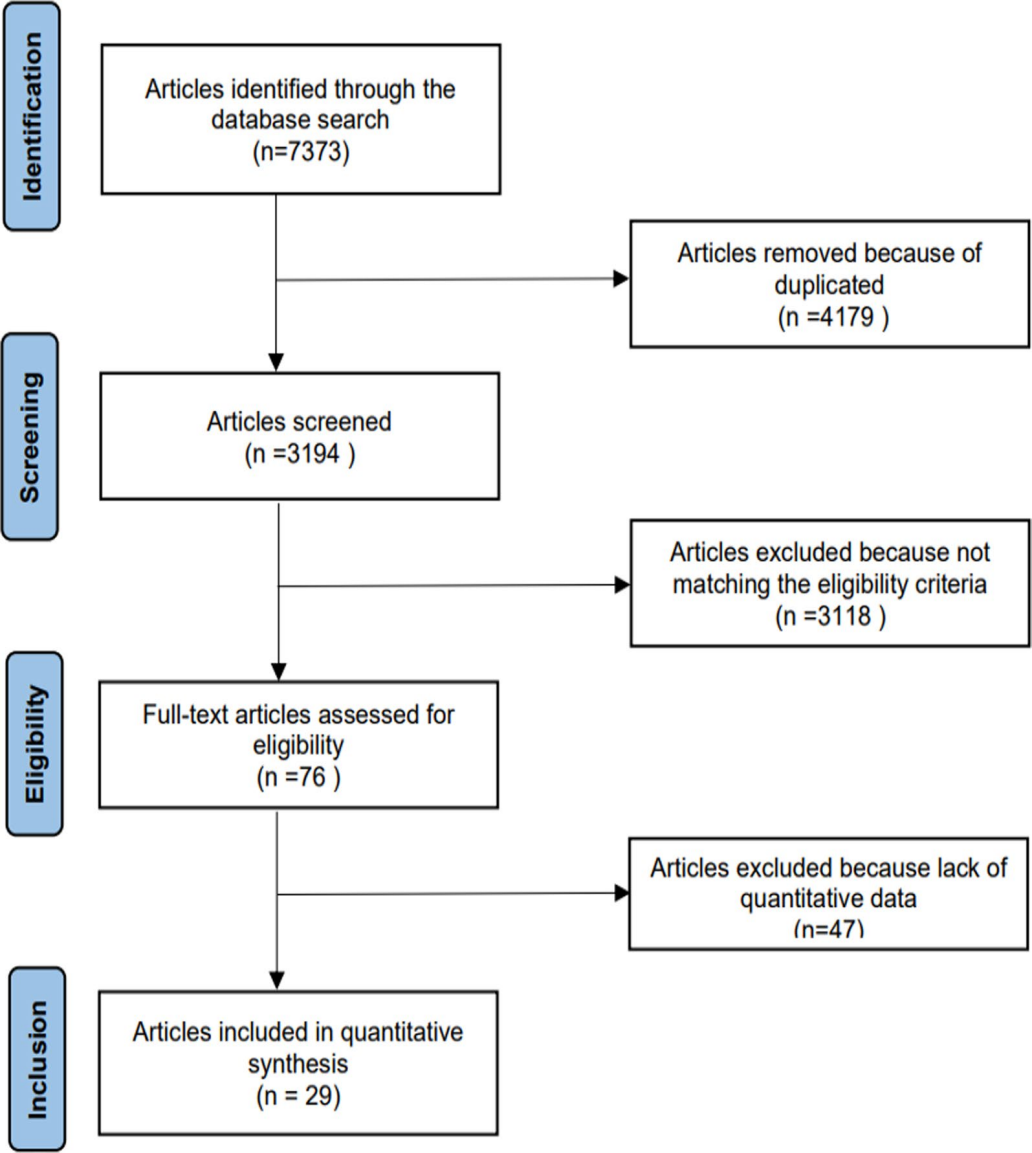
Procedure of literature enrollment

After searching, a total of 7373 articles were obtained. After the layer-by-layer screening, 29 studies [17–45] were finally included, with a total of 85,944 subjects. The literature screening process and results are shown in Fig. 2.

### Basic characteristics and quality assessment results of included studies

The essential characteristics of the included studies are shown in Table 2. The quality evaluation results of the included studies were as follows: there is 1 article [17] of one point, 7 [18, 21, 22, 24, 36, 41, 42] articles of two points, 13 articles [19, 20, 23, 27, 29–32, 35, 40, 43–45] of three points, 5 articles [28, 33, 37–39] of four points, and 3 articles [25, 26, 34] of five points. (See Additional file 1 for details).

**Table 2:** Basic characteristics of included studies

References	Region	OP assessment methods	Sample source	Position	Case size	Sample size	Prevalence (%)	Literature quality rating
Xia et al. [17]	China	BMD, X-ray	Postmenopausal women	Spine	388	2634	14.73	1
Lo et al. [18]	Hong Kong	BMD, X-ray	Random sampling women	Spine	113	1800	6.30	2
Zhang et al. [19]	Taiyuan	–	Postmenopausal women	Spine	38	614	23.17	3
Chai et al. [20]	Taiyuan	BMD	Postmenopausal women	Spine	40	195	20.51	3
Ju et al. [21]	Shanghai	BMD, X-ray	Random sampling population	Spine	204	4671	4.40	2
Cui et al. [24]	Beijing	BMD, X-ray, Bone metabolism examination	Postmenopausal women	Spine	337	1149	29.32	2
Gao et al. [22]	Shanghai	BMD, Related imaging examination	Random sampling population	Spine	2414	14,075	17.15	2
Chen et al. [23]	Shanghai	X-ray	Health examination population	Spine	323	510	63.33	3
Ma et al. [25]	Yinchuan	–	Inpatient	–	235	475	51.58	5
Chen et al. [26]	Haikou	BMD	Inpatient	–	165	339	48.67	5
Chen et al. [27]	Chongqing	BMD, Bone metabolism examination	Random sampling population	Hip, distal forearm, spine, and other	127	1000	12.70	3
Xu et al. [28]	Nanchang	BMD	Inpatient	Hip, distal forearm, spine, and other	382	3167	12.06	4
Li et al. [29]	Chengdu	BMD	Health examination population	Hip, distal forearm, spine, and other	468	1600	29.30	3
Wu et al. [30]	Shenzhen	–	Random sampling population	Hip, distal forearm, spine, and other	34	300	11.30	3
Yang et al. [31]	Guiyang	BMD, X-ray	Health examination population	spine	120	507	23.67	3
Chen et al. [32]	Guangzhou	–	Random sampling population	–	133	429	31.00	3
Wang et al. [33]	Chongqing	–	Inpatient	spine	264	996	26.50	5
Wang et al. [34]	Changchun	–	Inpatient	Hip, distal forearm, spine, and other	977	12,601	7.75	4
Chen et al. [35]	Haikou	BMD	Random sampling population	Hip, distal forearm, spine	51	531	9.60	3
Zhang et al. [36]	Hebei Province	BMD	Random sampling population	Hip, distal forearm, spine	124	1262	19.36	2
Xie et al. [37]	Zhaotong	Related imaging examination	Inpatient	Hip, spine, and other	654	4837	13.52	4
Ma et al. [38]	Xishuangbann-an	Related imaging examination	Inpatient	Hip, spine, and other	485	6021	8.06	4
Zhao et al. [39]	Kunming	Related imaging examination	Inpatient	Hip, spine, and other	563	3720	15.13	4
Zhang et al. [40]	Quanzhou	BMD, X-ray	Random sampling population	Hip, distal forearm, spine, and other	147	500	29.40	3
Zhu et al. [41]	Shanghai	Questionnaire survey, Health file	Random sampling population	Hip, distal forearm, spine, and other	1910	12,000	15.92	2
Liu et al. [42]	Yunnan Province	–	Random sampling population	–	402	6477	6.21	2
An et al. [43]	Chengdu	BMD, X-ray	Random sampling population	Spine	137	644	21.27	3
Shen et al. [44]	Wuhan	BMD, X-ray	Health examination population	Hip, distal forearm, spine, and other	129	1764	7.31	3
Xu et al. [45]	Guangzhou	X-ray	Random sampling population	Hip, distal forearm, spine, and other	137	1126	12.20	3

### Meta-analysis results

**Fig. 3:**
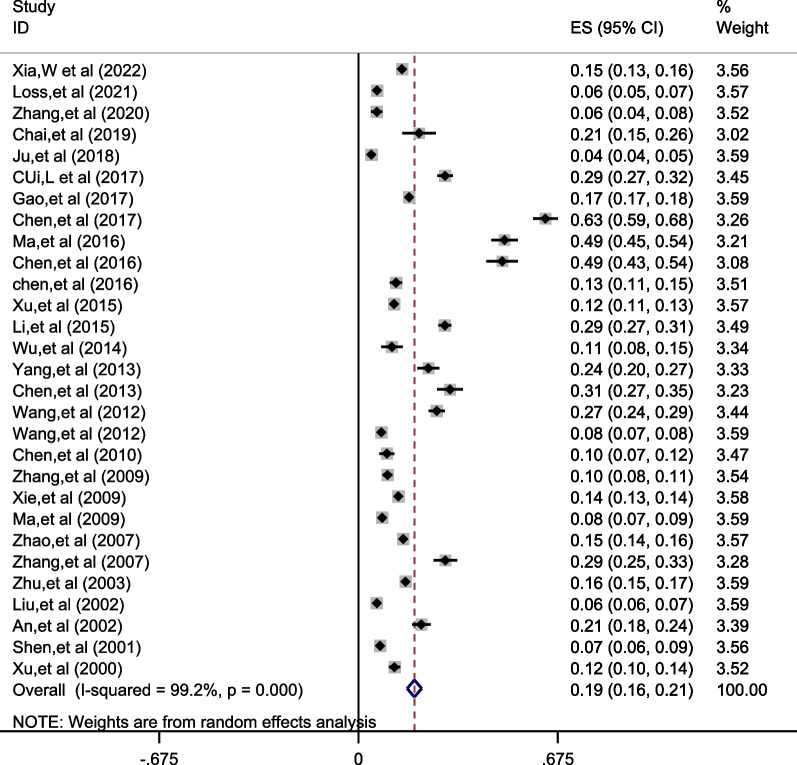
Meta-analysis forest plot of the total prevalence of osteoporotic fractures in older people in China

A total of 29 studies involving 85,944 older adults were included in this study. After meta-analysis, the prevalence of osteoporotic fractures in older people in China was 18.9% [95% CI (16.5%, 21.4%)] (See Fig. 3).

### Subgroup analysis

In this study, we set five subgroups: region (South, North)—Qinling-Huaihe as the boundary, north of Qinling-Huaihe as the north, south as the south, gender (men and women), age group (60–69, 70–79, ≥ 80), publication time (2000–2010, 2012–2022), and fracture site (vertebra, hip, distal forearm, and others). The specific results of subgroup analysis are shown in Table 3.

**Table 3:** Summary of meta-analysis results

Subgroup	Number of included studies	Results of heterogeneity test		Effects models	Prevalence rate % (95% CI)
		I^2^	*p*		
Total prevalence rate [17–45]	29	99.2%	< 0.001	Random effect model	18.9 (16.5, 21.4)
*Sex*					
Men [21–23, 27, 29, 30, 35, 36, 40–42, 44]	12	99.0%	< 0.001	Random effect model	14.3 (10.1, 18.5)
Women [17–20, 24–26, 28, 31–34, 37–39, 43, 45]	17	99.0%	< 0.001	Random effect model	18.5 (14.8, 22.3)
*Age*					
60–69 [19, 20, 23, 24, 26, 27, 29–32, 35, 36, 40–44]	17	98.0%	< 0.001	Random effect model	15.9 (12.2, 19.6)
70–79 [20, 23, 24, 26, 27, 29–32, 35, 36, 40–44]	16	98.4%	< 0.001	Random effect model	25.0 (19.6, 30.5)
≥ 80 [20, 23, 24, 26, 27, 29–32, 35, 36, 40–44]	16	98.5%	< 0.001	Random effect model	35.6 (27.9, 43.4)
*Area*					
South [17, 18, 21–23, 26–32, 34, 35, 37–45]	22	99.3%	< 0.001	Random effect model	18.9 (15.9, 21.8)
North [19, 20, 24, 25, 33, 36]	6	99.2%	< 0.001	Random effect model	20.3 (12.2, 28.3)
*Published time*					
2000–2010 [35–45]	11	98.7%	< 0.001	Random effect model	13.2 (10.4, 16.1)
2012–2022 [17–34]	18	99.4%	< 0.001	Random effect model	22.7 (18.7, 26.6)
*Position of fracture*					
Hip [27–30, 33, 35–41, 44, 45]	14	98.5%	< 0.001	Random effect model	4.5 (3.3, 5.7)
Proximal-forearm [27–30, 33, 35, 36, 40, 41, 44, 45]	11	94.9%	< 0.001	Random effect model	3.0 (2.2, 3.7)
Spine [18–24, 27–31, 33–41, 43–45]	25	99.6%	< 0.001	Random effect model	11.6 (9.8, 13.4)

### Sensitivity analysis

**Fig. 4:**
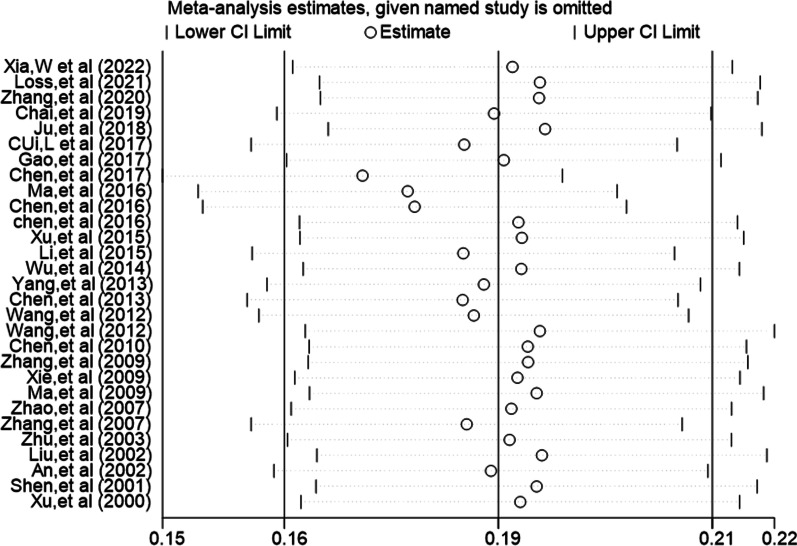
Included in the literature sensitivity analysis chart

We used the method of sequentially eliminating individual studies and recombining the total effect size to analyze the sensitivity of the total prevalence. The results showed that the majority of osteoporotic fractures in the elderly in China was between 16.0 and 21.0%, and there was no directional degeneration in each development, suggesting that the research results were relatively stable (See Fig. 4).

## Discussion

Our results show that osteoporotic fractures in the elderly in China have the following characteristics: First, the prevalence rate of osteoporotic fractures in women is higher than that in men in the same age group. Secondly, according to age, the prevalence rate of people over 80 years old is the highest, followed by 70–79 years old, and finally 60–69 years old. Third, the prevalence rate in the north is higher than that in the south. Fourth, the prevalence of osteoporotic fractures has increased significantly in the past decade (from 13.2% in 2000–2010 to 22.7% in 2012–2022). Fifth, the common sites of osteoporotic fractures are the spine, hip, and distal forearm. Finally, the prevalence of osteoporotic fractures among the Chinese elderly is high (18.9%), and the related prevention and treatment should not be relaxed.

The root cause of osteoporotic fracture lies in osteoporosis. Osteoporosis can lead to the destruction of bone fine structure, the decrease in bone mass, and the decrease in bone strength. it is very easy to cause a fracture when exposed to slight external force in daily life. One of the main risk factors for osteoporosis is gender, which is an unalterable factor. According to a comprehensive study in Iraq, the prevalence of osteoporosis is 12% in men, 3% in premenopausal women, and 19% in postmenopausal women, indicating significant gender differences [46]. There are similar results in the USA (4.5% vs 15.4%) [47]. The reason for this difference may be related to the rapid bone loss caused by the rapid decline of ovarian function and the decrease in estrogen levels in postmenopausal women. The longer the time of menopause, the more obvious the decrease in bone mineral density, resulting in osteopenia and further development of osteoporosis, increasing the risk of osteoporotic fracture [39]. Previous studies have shown that women lose about 55% of their body bone mass in their lifetime, while men lose about 35% of their body bone mass [48]. Gender is an important factor leading to osteoporosis and osteoporotic fracture. Women can take menopause as an important time point for the prevention and treatment of osteoporotic fractures and intervene in time. Aging is another unchangeable factor, and the South Korean National Health and Nutrition Survey (KNHANES) shows a significant age difference [49]. A large-scale survey in Austria shows that the prevalence of osteoporosis increases with age [50]. A cohort study showed that young participants diagnosed with osteoporosis had a higher prevalence of osteoporosis than older participants (35% vs 10.0%). It has been recognized that bone mineral density (BMD) decreases with age after reaching the optimal value. A clinical study involving 17,083 subjects showed that bone mass reduction rates in women aged 50–64 and ≥ 65 years old were 31% and 62%, respectively [51]. Our research is consistent with these studies. The region is also an unchangeable factor. The prevalence rate in the north is higher than that in the south, which is consistent with some previous studies. The prevalence of osteoporosis in northern Iraq is higher than that in southern Iraq, and the regional differences are due to differences in vitamin D levels. It is reported that vitamin D3 synthesis may not be sufficient to explain the decrease in BMD due to the lack of ultraviolet light at high latitudes [52]. In addition, the climatic environment may also affect the prevalence of osteoporotic fractures to some extent. The climate in the north is cold, and the roads are icy. In a cold environment, people's clothing will be thicker, physical flexibility will decrease, coupled with road icing, slippery roads, and other factors, will increase the chance of fall injury, leading to an increase in the prevalence of fractures. However, the specific mechanism behind this difference is not clear. The common sites of osteoporotic fractures were the spine (11.6%), hip (4.5%), and distal forearm (3.0%). The location of fracture is mainly related to physiological factors, specific anatomical location, and stress mode. Previous studies have also shown that [3]: the common sites of osteoporotic fractures are vertebrae (thoracic and lumbar vertebrae), hip (proximal femur), distal forearm, and other key parts, such as ribs, fibula, and other parts. In addition, in the past 10 years, the prevalence rate of osteoporotic fractures in the elderly in China has been on the rise. From 13.2% in 2000–2010 to 22.7% in 2012–2022, this difference may be caused by differences in health and medical resources between the past and the present.

Most of the diagnostic criteria for osteoporotic fractures included in the study were “osteoporosis + fracture-related diagnosis”. BMD based on DXA is the current “gold standard” for diagnosing osteoporosis, but DXA can be measured at the lumbar spine, proximal femur, or left forearm (non-dominant distal radius 1/3) [53]. Due to the different conditions of bone loss in different parts, the BMD measurement results were significantly different [54]. This condition may affect the prevalence of osteoporosis and osteoporotic fractures. In this study, we also noted that some of the included studies supplemented the detection of bone metabolic markers in the diagnosis of osteoporotic fractures. Relevant studies have shown that some bone metabolic markers (BTM) have great potential in monitoring the degree of osteoporosis progression [55, 56].

At present, according to China's Seventh National population Census [57]: China has 264 million people over 60 (about 18.7% of the total population), and more than 190 million people over 65 (about 13.5% of the total population), making it the country with the largest elderly population in the world. In addition, the relevant osteoporosis epidemiological survey showed that [58]: the prevalence rate of osteoporosis was 19.2% in people over 50 years old, including 32.1% in women and 6.9% in men. The prevalence rate of osteoporosis in people over 65 years old is 32.0%, including 51.6% in women and 10.7% in men. The prevalence rate of osteoporosis in women (32.1% over 50 years old) was significantly higher than that in Europe, America, Japan, and South Korea (16.5% in the USA, 15.8% in Canada, and 38.0% in South Korea). At present, osteoporosis has become the third largest chronic disease harmful to human health after cardiovascular disease and diabetes. Osteoporotic fractures are a disease of fundamental importance prevalent in global health systems, and their incidence is minimized by proper management [59]. Given the current situation in China, proactive prevention and control measures are needed to raise citizens' awareness of the situation through a three-step prevention plan. In addition, it is also necessary to actively evaluate the efficacy and safety of anti-osteoporosis drugs, which can refer to relevant foreign studies [60, 61]. Timely intervention and treatment of osteoporosis are the key to prevent osteoporotic fractures.

This is the first systematic review and meta-analysis of the prevalence of osteoporotic fractures in the elderly in China, and relevant prevalence information is the first step in developing preventive procedures or health care services for the elderly population. In addition, the relevant data obtained in our study have many potential benefits in clinical application, which need to be further explored such as screening for the disease, recommended screening tools, and so on. We searched in detail through multiple databases to avoid missing important evidence. Included studies were analyzed using a standardized process, and included studies were evaluated for quality, heterogeneity, sensitivity, etc. Subgroups were set up based on sex, age, region, publication time, fracture site, and subgroup analysis to gain more insight into the possible causes of heterogeneity between studies. The study also has some limitations. First, we collected representative data for each region but differed in terms of sample source, sample size, diagnostic criteria, etc., which may affect comparisons between included studies. Secondly, the heterogeneity of the included studies was high, and the source of heterogeneity could not be found through sensitivity analysis and subgroup analysis. Third, the number of groups is relatively small, and the representativeness may be affected to some extent.

Future studies should mitigate the degree of heterogeneity by conducting additional subgroups to investigate other relevant risk factors. Secondly, subgroup analysis does not imply any causal variables. Therefore, more studies need to be recruited from longitudinal follow-up studies and use meta regression techniques to identify predictors of osteoporotic fractures. Finally, further research should also investigate osteoporotic fractures in middle-aged adults to accelerate the design and implementation of targeted therapies to prevent or mitigate the progression of osteoporotic fractures in community and clinical settings.

## Conclusion

In the past 10 years, the prevalence of osteoporotic fractures in the older age group ≥ 60 has increased in China. The prevalence of osteoporotic fractures increases with age, higher in women than in men and higher in the north than in the south. With the increasing prevalence of osteoporotic fractures, the relevant prevention and control measures in the health sector become more important.

## Supplementary Information

**Additional file 1.** Basic characteristics of included studies.

## Data Availability

All data generated or analyzed during this study are included in this published article.
